# Hope Modified the Association between Distress and Incidence of Self-Perceived Medical Errors among Practicing Physicians: Prospective Cohort Study

**DOI:** 10.1371/journal.pone.0035585

**Published:** 2012-04-18

**Authors:** Yasuaki Hayashino, Makiko Utsugi-Ozaki, Mitchell D. Feldman, Shunichi Fukuhara

**Affiliations:** 1 Department of Epidemiology and Healthcare Research, Kyoto University Graduate School of Medicine and Public Health, Kyoto, Japan; 2 Horikawa Hospital, Kyoto, Japan; 3 Department of Medicine, University of California San Francisco, San Francisco, California, United States of America; Federal University of Rio de Janeiro, Brazil

## Abstract

The presence of hope has been found to influence an individual's ability to cope with stressful situations. The objective of this study is to evaluate the relationship between medical errors, hope and burnout among practicing physicians using validated metrics. Prospective cohort study was conducted among hospital based physicians practicing in Japan (N = 836). Measures included the validated Burnout Scale, self-assessment of medical errors and Herth Hope Index (HHI). The main outcome measure was the frequency of self-perceived medical errors, and Poisson regression analysis was used to evaluate the association between hope and medical error. A total of 361 errors were reported in 836 physician-years. We observed a significant association between hope and self-report of medical errors. Compared with the lowest tertile category of HHI, incidence rate ratios (IRRs) of self-perceived medical errors of physicians in the highest category were 0.44 (95%CI, 0.34 to 0.58) and 0.54 (95%CI, 0.42 to 0.70) respectively, for the 2^nd^ and 3^rd^ tertile. In stratified analysis by hope score, among physicians with a low hope score, those who experienced higher burnout reported higher incidence of errors; physicians with high hope scores did not report high incidences of errors, even if they experienced high burnout. Self-perceived medical errors showed a strong association with physicians' hope, and hope modified the association between physicians' burnout and self-perceived medical errors.

## Introduction

Medical errors and patient safety continue to be important concerns for both patients and physicians, especially since the Institute of Medicine report (1999) that between 48,000 and 98,000 Americans die each year due to preventable adverse events [1]. In Japan, 1,010 adverse drug events and 514 medication errors (incidence: 17.0 and 8.7 per 1,000 patient-days, respectively) were observed over 6 months in a cohort of 3,459 adults admitted to tertiary care hospitals [2]. It has been estimated that up to 50% of hospitalized patients are affected by medical errors [3], [4] with high human and monetary costs [5]–[9]. Additionally, it has been reported that burnout and depression may undermine the quality of care and contribute to medical errors [10]–[12], but the individual factors contributing to these errors have not been adequately evaluated.

Hope has been described as the belief that the present situation can be modified and that there is a way out of difficult situations or the belief that better days or moments will come [13]. Dufault and Martocchio define hope as “a multidimensional dynamic life force that is characterized by a confident yet uncertain expectation of achieving good, which is realistically possible and personally significant" [14]. Hope is an important personal resource that influences an individual's ability to cope with stressful, life-threatening situations [15]–[17]. It is thus reasonable to hypothesize that hope attenuates the association between physicians' distress and medical errors by influencing the ability of physicians to cope with difficult clinical situations. The primary objective of this study is to evaluate whether hope modifies the association between physician burnout and medical errors using validated metric. We secondarily evaluate whether physicians with high hope are less likely to make medical errors.

## Results

Of a total of 1198 subjects to whom we had sent the solicitation e-mails, 836 physicians (69.8%) participated in the baseline survey, and all of these physicians responded to the follow-up survey. The demographic characteristics of the study participants are given in Table 1. Baseline participant characteristics for burnout, depression screening, and hope, and reported self-perceived medical errors during follow-ups are given in Table 2. Overall, 183 study participants (21.9%) reported at least 1 major medical error during the past year of the survey, and a total of 361 errors were reported in 836 physician-years.

**Table 1:** Participating physicians' characteristics, 2009.

Variable	n = 836
Age, n (%)	
–39	191 (22.9)
40–49	390 (46.7)
50–59	221 (26.4)
60–	34 (4.0)
Male, %	92.1
Specialty, %	
Generalist	18.8
Specialized internist	26.1
Pediatrician	8.5
Surgeon	35.5
Other	11.1

**Table 2:** Self-reported medical errors, burnout, and depression among male and female practicing physicians.

Variable	Participants and scores
**Burnout**	
Mean emotional exhaustion score (SD) [range]	13.5 (4.0) [5–25]
Mean depersonalization score (SD) [range]	13.2 (4.7) [6–30]
Mean personal accomplishment score (SD) [range]	17.5 (4.4) [6–30]
**Depressive symptoms, n (%)**	
WHO-5* score <13 (screen positive)	453 (54.2)
WHO-5* short version score ≥13 (screen negative)	383 (45.8)
**Hope**	
Mean Herth Hope Index score (SD) [range]	34.2 (5.6) [12–48]
**Medical errors**	
Physicians who reported at least 1 error, n (%)	183 (21.9)
Mean number of self-reported medical errors (SD) [range]	0.43 (1.9) [0–50]

* WHO-5, World Health Organization-Five Well-being Index.

Summary measures to identify general associations between self-perceived errors and physicians' burnout, and symptoms of depression, and hope, are given in Table 3. We observed a significant univariate association between HHI score tertiles and self-perceived medical errors (p = 0.034). Physicians reporting at least one error during the study period had significantly higher levels of burnout, as evidenced by increased EE level tertiles (p = 0.026) and increased DP level tertiles (p = 0.002). We did not observe a significant association in the PA score (p = 0.668) and depression (p = 0.058) with reporting at least 1 medical error.

**Table 3:** Baseline characteristics and medical errors during follow-ups among physicians with and without self-reported medical errors among practicing male and female physicians.

Variable	All	Any medical error	No medical error	No. of reported errors		
	n = 836	n = 183	n = 653	No.	Mean	p-value†
**Age, n (%)**						0.647
28–39	191 (22.9)	48 (26.2)	134 (21.9)	136	0.71	
40–49	390 (70.7)	80 (43.7)	310 (47.5)	136	0.35	
50–59	221 (26.4)	48 (26.2)	173 (26.5)	79	0.36	
60–81	34 (4.1)	7 (3.8)	27 (4.1)	10	0.29	
**Sex, n (%)**						0.008
Male	770 (92.1)	177 (96.7)	593 (90.8)	354	0.46	
Female	70 (739)	6 (3.3)	60 (9.2)	7	0.10	
**Burnout, n (%)**						
Emotional exhaustion score (score range)						0.026
1st tertile (5–11)	286 (34.2)	51 (27.9)	235 (36.0)	79	0.28	
2nd tertile (12–15)	310 (37.1)	70 (38.2)	240 (36.8)	113	0.36	
3rd tertile (16–25)	240 (28.7)	62 (33.9)	178 (27.2)	169	0.70	
Depersonalization score (score range)						0.002
1st tertile (6–11)	346 (41.4)	64 (35.0)	282 (43.2)	91	0.26	
2nd tertile (12–15)	264 (31.6)	51 (27.8)	213 (32.62)	95	0.36	
3rd tertile (16–30)	226 (27.0)	68 (37.2)	158 (24.2)	175	0.77	
Personal accomplishment score (score range)						0.668
1st tertile (6–16)	334 (40.0)	74 (40.0)	260 (39.8)	183	0.55	
2nd tertile (17–20)	277 (33.1)	63 (34.4)	214 (32.8)	105	0.38	
3rd tertile (21–30)	225 (26.9)	46 (25.1)	179 (27.4)	73	0.32	
**Depressive symptoms, n (%)** *						0.058
WHO-5 score ≥13 (screen negative)	383 (4538)	74 (40.4)	309 (47.3)	383	0.31	
WHO-5 score <13 (screen positive)	453 (54.2)	109 (59.6)	344 (52.7)	453	0.53	
**Hope**						0.034
1st tertile (12–33)	320 (38.3)	83 (45.4)	237 (36.3)	202	0.63	
2nd tertile (34–36)	264 (31.6)	45 (24.6)	219 (33.5)	74	0.28	
3rd tertile (37–48)	252 (30.1)	55 (30.0)	197 (30.2)	85	0.34	

* WHO-5, World Health Organization-Five Well-being Index.

† Fisher's exact test or trend test between any medical error and no error.

The age- and sex-adjusted association between burnout, depression, or hope and the number of self-perceived medical errors is given in Table 4. There was a significant association between the HHI score tertiles, and error reporting compared with the lowest tertile category of HHI; IRRs of self-perceived medical errors of physicians were 0.44 (95%CI, 0.34 to 0.58) and 0.54 (95%CI, 0.42 to 0.70) respectively, for the 2^nd^ and 3^rd^ tertiles. We observed a significant association between the burnout EE, DP and PA domain scores, and error reporting; compared with the lowest tertile category, IRRs of self-perceived medical errors of physicians in the highest category were 2.34 (95%CI, 1.16 to 1.68: p<0.0001) 2.72 (95%CI, 1.15 to 1.63: p<0.0001), and 0.62 (95%CI, 0.47 to 0.82: p = 0.0001) respectively, for EE, DP and PA. Depression was also associated with self-perceived medical errors of physicians 1.67 (95%CI, 1.34 to 2.09: p<0.0001). We further explored the association between hope, burnout, and medical error. HHI score was associated with medical errors. HHI score was significantly associated with burnout EE score (correlation coefficient −0.4434, p<0.0001), DP score (correlation coefficient −0.4810, p<0.0001), and PA score (correlation coefficient 0.5339, p<0.0001). The association between HHI score and medical error was still significant in the multivariable-adjusted model containing EE score (p for trend <0.0001), DP score (p for trend <0.0001), or PA score (p for trend 0.011).

**Table 4:** Relationship between burnout, depression, or hope and incidence of self-reported medical errors among male and female practicing physicians, 2009.

Variable	Adjusted IRR (95%CI)†	P value
**Burnout**		
Emotional exhaustion score tertiles (score range)		
1st (5–11)	Reference	
2nd (12–15)	1.28 (0.88 to 1.28)	0.096
3rd (16–25)	2.34 (1.16 to 1.68)	<0.0001
Depersonalization score tertiles (score range)		
1st (6–11)	Reference	
2nd (12–15)	1.28 (0.80 to 1.16)	0.096
3rd (16–30)	2.72 (1.15 to 1.63)	<0.0001
Personal accomplishment score tertiles (score range)		
1st (6–16)	Reference	
2nd (17–20)	0.64 (0.50 to 0.81)	<0.0001
3rd (21–30)	0.62 (0.47 to 0.82)	0.001
**Depression** *		
WHO-5 score <13 (screen positive)	Reference	
WHO-5 score ≥13 (screen negative)	1.67 (1.34 to 2.09)	<0.0001
**Hope**		
Herth Hope Index score tertiles (score range)		
1st (6–16)	Reference	
2nd (17–20)	0.44 (0.34 to 0.58)	<0.0001
3rd (21–30)	0.54 (0.42 to 0.70)	<0.0001

* WHO-5, World Health Organization-Five Well-being Index.

† **I**RR, incidence rate ratio adjusted for age and sex.

**Figure 1:**
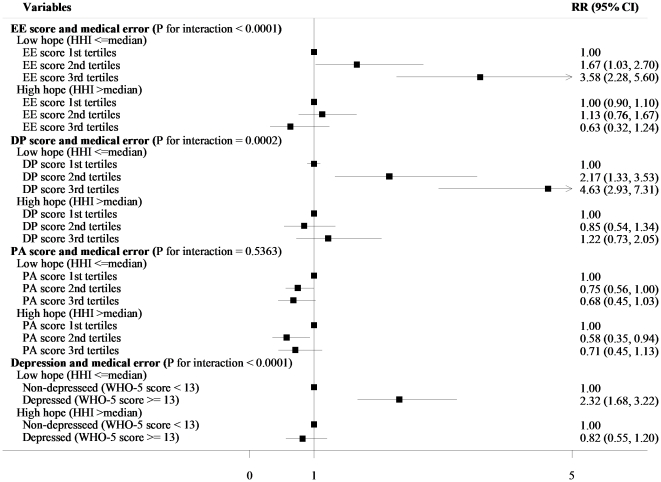
Association between burnout and medical errors: subgroup analysis by depressive symptoms evaluated by WHO-5 or Herth Hope Index score among practicing physicians, males and females, 2009. WHO-5, World Health Organization-Five Well-being Index; HHI, Herth Hope Index; IRR, Incidence rate ratio adjusted for age and sex.

Finally, the association between each item of the Burnout Scale (EE, DP, PA), or depression and self-perceived medical errors of physicians stratified by HHI score is given in Figure 1. We observed a statistically significant interaction between HHI and Burnout Scale EE (P for interaction <0.0001) or DP (P for interaction = 0.0002) and for reporting errors. Among physicians with a low hope score, those who experienced higher burnout reported a higher incidence of errors; compared with the lowest tertile category, IRRs of self-perceived medical errors of physicians in the highest category were 3.58 (95%CI, 2.28 to 5.60) and 4.63 (95%CI, 2.93 to 7.31), respectively, for EE and DP. We did not observe a significant interaction between burnout PA score and HHI (P for interaction = 0.5363). We observed the interaction between depression and HHI score (P for interaction <0.0001); among physicians with a low hope score, depressed physicians reported higher incidence of errors (IRR 2.32; 95%CI, 1.68 to 3.22), while depressed physicians did not report higher incidence of errors if they reported higher HHI score (IRR 0.82; 95%CI, 0.55 to 1.20).

## Discussion

Our study is the first to show that physicians who have low levels of hope are more likely to report self-perceived errors than those with average and high levels of hope. We also found that hope worked as an effect modifier of the known association between physicians' distress and self-perceived medical errors. A possible explanation for our findings is that hope decreased the reporting of self-perceived medical errors by mediating the psychological distress that usually accompanies a mistake. Prior research has found that those with higher levels of hope reported lower levels of psychological distress and a better QOL [18], and these have been proven to be factors that are associated with a low incidence of self-perceived medical errors [10]. For example, some physicians who have higher levels of hope, even though they rate high on the burnout scale, may see possibilities for the future, and as a result, their psychological distress may be reduced. In contrast, physicians with lower levels of hope are not able to adjust to a difficult work environment, and report higher frequency of medical errors.

Hope is usually researched in relation to terminal care [19]–[22] and in such situations hope is usually treated as a better outcome, not an exposure. Jerome Groopman suggests in his book, The Anatomy of Hope, that “to have hope, then is to acquire a belief in your ability to have some control over your circumstances"; this assertion is supported by our results [23]. One study that evaluated hope as an effect-modifying factor supports our hypothesis. Among 194 female patients with breast cancer, hope measured by HHI was found to mediate the relationship between psychological distress and health status, such that the direct association between distress and health status was no longer significant after hope was included as a variable [18].

We found that self-perceived major medical errors were common among the practicing physicians, with approximately one-third of the participants reporting a major error at least once during 1 year. Our result that physicians commonly report self-perceived major medical errors is consistent with a previous report. In a survey of 184 resident physicians, an average of 14.7% of participants reported errors per resident-quarter [11]. In a cross-sectional study among 7905 members of the American College of Physicians, 700 (7.9%) physicians reported a concern they had made a major medical error in the previous 3 months [10].

Most previous studies on errors among practicing physicians focus on systemic issues that contribute to errors rather than on individual-level distress factors [24]–[28]. Consistent with this focus, efforts to reduce errors have largely centered on improving coordination of care, teamwork, electronic order systems, and other system-related changes [24]–[30]. However, it has been postulated that many explanatory factors for medical errors “remain to be uncovered" [31]. Efforts to reduce errors resulting from individual-level distress factors, as we suggested, need to incorporate a variety of strategies, including efforts to reduce physician's degree of emotional distress and burnout [25]. Not only reducing emotional distress, our results suggest that the strategy to increase hope among physicians might directly and indirectly reduce medical errors, although this needs to be tested in a prospective intervention study.

This study has some limitations. First, because this is an observational study, residual confounding might explain our results. Especially, we asked about medical errors in the past 12 months, which differs from the 3 months in the previous studies [10], [11]. Using longer time frame might allow other factors such as life events and changes in fatigue to confound our results. Proportion of female physician of our sample was very low, although this is typical in Japanese physician population. Our results might not be generalizable to other non-Japanese physician population. In addition, HHI is a validated metrics to measure hope among patients, it has not been formally validated among physicians. Although this tool has never been used in physicians, this tool is used to measure hope in other health professional (nurses) [32]. In addition, we did not observe ceiling effect or flooring effect in our sample. And more, our study suggested that physicians' hope measured by HHI discriminate between high error rate and low. For these reasons, we believe HHI is useful among physicians.

In conclusion, physicians' hope modified the association between physicians' burnout and self-perceived medical errors, and hope also exhibited a strong association with self-perceived medical errors. Efforts to reduce errors resulting from individual-level distress factors need to incorporate a strategy to increase physician's degree of hope.

## Materials and Methods

### Participants

The study participants were members of a panel of 6459 hospital based physicians organized by a web-based survey company (Plamed Co., Tokyo, Japan). These physicians were recruited through hospital lists or at scientific meetings. Subjects were aware that participation in the survey was completely voluntary. We obtained prior approval from the Research Ethics Committee of Kyoto University Graduate School of Medicine (E 970).

### Data Collection

An anonymous web-based baseline survey was conducted from November 17 to 24, 2009. Solicitation e-mails were sent to a random sample of 1198 physicians out of all 6549 registered physicians to request participation in the survey. The e-mails included a brief introduction describing the objectives of the study as well as statements guaranteeing confidentiality and anonymity of responses. Participants were given 7 days to complete the survey. During this time, physicians could log-in to the survey website using their membership identification and password and complete the survey without entering their name. Participants who participated in the baseline survey were asked to take a follow-up survey conducted 1 year later. The participants were offered 1000 Japanese yen (approximately US$13) gift card upon completion of the survey.

### Study Measures

Surveys included questions about demographic characteristics, specialty, and report of self-perceived medical errors. Validated survey tools were used to measure burnout, symptoms of depression, and hope.

The Japanese version of the Herth Hope Index (HHI) was used to assess “hope" [33]. The measure is a 12-item (1–4 point) Likert scale investigating general sense of psychosocial strength or positive expectations, with items such as “I can see possibilities in the midst of difficulties" and “I feel my life has value and worth." The measure was derived from a previous more comprehensive instrument, the Herth Hope Scale. The total score can range from 12 to 48, with higher scores denoting greater hope. Previous research supports this measure as a reliable and valid correlate of successful adjustment to the illness experience and maintenance of a sense of well-being [34]–[37]. The Japanese version of HHI, which has good construct validity and reliability, was used in a previous study [38].

Perceived medical errors were evaluated at the follow-up survey in 2010 by asking the question, “Are you concerned that you have made any major medical mistakes in the last year?" This question was used in a previous study that evaluated medical errors among resident physicians [10], [11]. If the response to this question was “yes," we then asked about the number of medical errors that concerned them. The intent of this question was to identify errors internalized by physicians, rather than to document events associated with patient risk. Thus, the self-reported errors in this study represent major medical errors as perceived by each physician [10].

Burnout is a syndrome encompassing 3 domains (depersonalization (DP), emotional exhaustion (EE), and a sense of low personal accomplishment (PA)) that are associated with decreased work performance [39]. Burnout was measured using the 17-item Burnout Scale based on the Maslach Burnout Inventory (MBI), developed by Tao et al. [40] specifically for Japanese healthcare professionals. The Burnout Scale consists of three subscales: DP (six items), EE (five items), and PA (six items). Participants were asked to rate the frequency with which they experience various feelings or emotions on a 5-point Likert scale. This scale has good internal consistency and convergent validity [41]–[45]. Higher values of DP and EE and lower values of PA signify burnout. Developers of this scale recommend that this tool should be used to compare relative degree of burnout at this time, but conventionally set the threshold of burnout as > = 18, > = 21, and > = 16, respectively, for DP, EE, and PA. This instrument has been used in numerous previous studies of health professionals [41]–[45].

Depression was assessed with the World Health Organization-Five Well-Being Index (WHO-5), which is designed to assess the absence of positive mood using five items on a 6-point frequency scale [46]. WHO-5 attempts to establish a score that measures the extent to which a person has experienced positive well-being in the two weeks prior to assessment [47], [48]. Although raw scores can vary between 0 and 25, lower scores demonstrate a poor overall feeling of well-being [49]. A cut-point of less than or equal to 13 has been proven to offer 94% sensitivity and 65% specificity as a diagnostic tool for depression [49] with acceptable findings for internal consistency (Cronbach's alpha, 0.83) [50]. For this study, the Japanese language version of the WHO-5 was used for all participants [49].

### Statistical Analyses

Standard univariate analysis was used to characterize the sample. Comparisons between physicians reporting errors and physicians reporting no errors were initially performed using summary statistics. These outcomes were analyzed using the Fisher's exact test for dichotomous proportions, or trend test for proportions with equal to or more than 3 categories.

The prospective association between burnout, depression, and hope at baseline and the number of self-perceived errors thereafter was evaluated using Poisson regression adjusting for age and sex, which is suited to counted data [51], to estimate the incidence rate ratios (IRRs) for medical errors and its 95% confidence intervals (CI). Multicollinearity among burnout, depression, and hope variables required that each model include self-reported errors and no more than 2 of these variables. For this analysis, each item of the Burnout Scale (DP, EE, and PA) was categorized into tertiles, and HHI was categorized as a dichotomous variable (≤median or >median). In addition, we constructed a statistical model which include HHI score and each of burnout score (EE, DP, PA) to explore the association between hope, burnout, and medical error.

Next, we evaluated whether hope modified the association between Burnout Scale (DP, EE, PA), or depression and self-perceived medical error. The presence of interaction was also tested by adding a cross-product term between hope and burnout or depression to a Poisson regression model adjusted for age (at baseline) and sex. Statistical significance was set at 0.05 level, and all tests were 2-tailed. All statistical analyses were performed using a statistical software package (STATA, version 11.0; STATA Corp; College Station, TX, USA).
